# Tailoring the Physico-Chemical Properties of Poly(xylitol-dicarboxylate-*co*-butylene dicarboxylate) Polyesters by Adjusting the Cross-Linking Time

**DOI:** 10.3390/polym12071493

**Published:** 2020-07-03

**Authors:** Marta Piątek-Hnat, Paulina Sładkiewicz, Kuba Bomba, Jakub Pęksiński, Agnieszka Kozłowska, Jacek G. Sośnicki, Tomasz J. Idzik

**Affiliations:** 1 Department of Polymer and Biomaterials Science, Faculty of Chemical Technology and Engineering, West Pomeranian University of Technology, Piastów Ave. 42, 71-065 Szczecin, Poland; paulina.sladkiewicz@gmail.com (P.S.); bk34688@zut.edu.pl (K.B.); agak@zut.edu.pl (A.K.); 2 Faculty of Electrical Engineering, West Pomeranian University of Technology, Sikorskiego Ave. 37, 71-313 Szczecin, Poland; jakub.peksinski@zut.edu.pl; 3 Department of Organic and Physical Chemistry, Faculty of Chemical Technology and Engineering, West Pomeranian University of Technology, Piastów Ave. 42, 71-065 Szczecin, Poland; jacek.sosnicki@zut.edu.pl (J.G.S.); tomasz.idzik@zut.edu.pl (T.J.I.)

**Keywords:** ester elastomers, renewable sources, crosslinking, mechanical properties, biodegradation

## Abstract

Determining the cross-linking time resulting in the best achievable properties in elastomers is a very important factor when considering their mass production. In this paper, five biodegradable polymers were synthesized—poly(xylitol-dicarboxylate-co-butylene dicarboxylate) polymers, based on xylitol obtained from renewable sources. Five different dicarboxylic acids with even numbers of carbon atoms in the aliphatic chain were used: succinic acid, adipic acid, suberic acid, sebacic acid, and dodecanedioic acid. Samples were taken directly after polycondensation (prepolymer samples) and at different stages of the cross-linking process. Physiochemical properties were determined by a gel fraction test, differential scanning calorimetry (DSC), Fourier-transform infrared spectroscopy (FTIR), quasi-static tensile tests, nuclear magnetic resonance spectroscopy (^1^H NMR and ^13^C NMR), and an in vitro biodegradation test. The best cross-linking time was determined to be 288h. Properties and degradation time can be tailored for specific applications by adjusting the dicarboxylic acid chain length.

## 1. Introduction

Due to environmental concerns and declines in world oil reserves, it is of great importance to find monomers for polymer synthesis that are obtainable from renewable sources. One such group of compounds is sugar alcohols. These multifunctional alcohols exhibit a great deal of flexibility in terms of their possible applications as monomers for polymer synthesis, with the most researched group of sugar alcohol-based polymers being poly(polyol dicarboxylate). In general, polymers belonging to this polyester family have been proven to be biocompatible both in vitro and in vivo [1,2,3]. While retaining a core property of biodegradability, and eco-friendliness, a wide variety of sugar alcohol-based elastomers with different physico-chemical properties suitable for specific uses can be obtained by using different dicarboxylic acids and a specific sugar alcohol, like erythritol [4] or xylitol [5]. Another way to fine tune the characteristics is to choose a specific dicarboxylic acid and perform the synthesis using different sugar alcohols [3,6] Further tailoring of their properties can be performed by copolymerization with various diol-dicarboxylates [7,8,9].

Research into specific possible applications of poly(polyol dicarboxylate) polyesters has been reported in the literature. Poly(glycerol sebacate) has attracted the largest amount of attention, with a wide range of potential uses including scaffolds for the regeneration of bone tissue [10,11], myocardial tissue [12], retinal cells [13], blood vessels [14], nerves [15], cartilage [16], and as drug carriers [17]. 

Potential medical uses of xylitol-based polymers have also been reported. They include poly(xylitol-co-maleate-co-PEG) hydrogels for cell encapsulation [18], and fibrous networks of poly(xylitol sebacate) electrospun using the core–shell method for potential tissue engineering applications [19,20].

In our previous work various elastomers were synthesized and tested: poly(sorbitol sebacate-co-butylene sebacate) [21] and poly(xylitol sebacate-co-butylene sebacate) [22]. Poly(polyol sebacate-co-butylene sebacate) elastomers were also proven to be suitable for radiation modification. [23] The influence of the cross-linking time on the physiochemical properties of elastomers synthesized using succinic and sebacic acid was also tested [24] The novelty of this work is performing a successful synthesis of sugar alcohol-based polyesters without the use of a catalyst and using a much wider range of dicarboxylic acids (consisting of two, four, six, eight and 10 carbon atoms in the aliphatic chain) as monomers.

## 2. Materials and Methods

### 2.1. Synthesis of Elastomers 

All reagents were purchased from Sigma-Aldrich (St. Louis, MO, USA). All the reagents were of over 99% purity. Synthesis of five polymers was carried out. The monomers used were: butanediol, xylitol and five different dicarboxylic acids with even-numbered chain lengths—succinic acid (poly(xylitol succinate-co-butylene succinate) (PXBSu), adipic acid (poly(xylitol adipicate-co-butylene adipicate)) (PXBA), suberic acid (poly(xylitol suberate-co-butylene suberate)) (PXBSb), sebacic acid (poly(xylitol sebacate-co-butylene sebacate)) (PXBS), and dodecanedioic acid (poly(xylitol dodecanedioate-co-butylene dodecanedioate)) (PXBD). A monomer ratio of 2:1:1 (dicarboxylic acid: xylitol:butylene glycol) was used. Esterification of the dicarboxylic acid, xylitol and butylene glycol in N_2_ atmosphere at 150 °C for 13.5h was the first step of each synthesis. The second step was polycondensation in a vacuum atmosphere at 150 °C. After the synthesis materials were cast into silicone forms and cross-linked at 100 °C in 100 mb in a vacuum dryer. Materials were considered fully cross-linked after 288h because the value of the gel fraction did not increase for the materials cross-linked for a longer period of time.

### 2.2. Experimental Methods

#### 2.2.1. Nuclear Magnetic Resonance Spectroscopy (NMR) 

^1^H and ^13^C NMR spectroscopic measurements were performed on a Bruker DPX 400 AVANCE III HD spectrometer (Bruker, Rheinstetten, Germany) operating at 400.1 and 100.6 MHz, respectively. Approximately 50 mg of each sample was dissolved in 0.7mL of deuterated chloroform (CDCl_3_). TMS was used as an internal reference and spectra were acquired in 5-mm probes. For NMR analyses, the MestReNova (version 12.0.3, Mestrelab, Santiago de Compostela, Spain) program was used.

#### 2.2.2. Fourier Transform Infrared Spectroscopy (FTIR)

An analysis of the chemical structure of the polymers was conducted with Fourier transform infrared spectroscopy (FTIR). An Alpha Spectrometer Bruker (Bruker, Germany) was used. Recorded transmission spectra were in the range between 4000 cm^−1^ and 400 cm^−1^ with resolution of 2 cm^−1^. In order to develop the results, Omnic 7.3 software by the Thermo Electron Corporation (Waltham, MA, USA) was used.

#### 2.2.3. Differential Scanning Calorimetry (DSC)

In order to determine the thermal properties of the materials, differential scanning calorimetry (DSC) was utilized. TA Instruments apparatus Q2500 (New Castle, DE, USA) was used. The parameters of the analysis were −90 °C to 150 °C heating cycle and 10 °C/min heating rate. Tests were performed in a nitrogen atmosphere. In order to develop the results, TA Instruments Universal Analysis 2000, 3.9a software (New Castle, DE, USA), was used. 

#### 2.2.4. Mechanical Properties

The testing of the mechanical properties was performed using an Instron 36 instrument (Norwood, MA, USA). The parameters of the tests were 25 °C, 50% relative humidity, 100 mm/min crosshead speed, and 500 N load cell. Tests were performed in keeping with PN-EN-ISO 526/1:1996 standard. In total, 12 samples were used for each test.

#### 2.2.5. Hardness

Hardness (H) for materials after 288 h was measured using a Zwick/Material Testing 3100 Shore A hardness tester.

#### 2.2.6. Gel Fraction

In order to determine the gel fraction, the PN-EN-579:2001 extraction method was utilized. Samples of material after 48 h, 192 h and 288 h (fully cross-linked material) were used. The weight of each sample was about 1 g. A Schott crucible Type P2 was utilized. Samples were extracted with 100 cm^3^ of boiling tetrahydrofuran. The extraction took 3 h. A vacuum oven was used to dry the samples. The drying process took 3 h and was performed at 25 °C. After that, the samples were dried in a desiccator. Three samples for each cross-linking time were used. Formula (1) was used in order to calculate the gel fraction content. The mean of three measurements was used.

(1) $$X=\frac{m_{1}}{m_{0}}\times 100\%$$

where *m*_0_—mass of the sample before extraction, *m*_1_—mass of the sample after extraction.

#### 2.2.7. Hydrolytic Degradation

Hydrolytic degradation was carried out on previously sterilized 10 mm polymer discs for 21 days in phosphate-buffered saline (PBS) (Sigma Aldrich, Poznań, Poland) (pH range 7.1–7.2), in 37 °C. Three samples were used. PBS solution was changed every 48 h. Sterilization was conducted in a laminar chamber for 15 min, using UV light. Samples were placed in a 24-well plate. Each sample was covered with 1.5 mL of the solution. Samples after degradation were dried in a vacuum dryer in 25 °C, and then weighed. Mass loss after 21 days was calculated using formula (2)

D = (m_0_ − m_1_)/m_0_ × 100% (2)

where D—mass loss [%], m_0_—sample mass before degradation [g], m_1_—sample mass after degradation [g].

#### 2.2.8. Enzymatic Degradation

Enzymatic degradation was carried out on previously sterilized 10-mm polymer discs for 21 days in solution of lipase (*Pseudomonas Cepacia*) (Sigma Aldrich, Poznań, Poland) (25 units/mL) in phosphate-buffered saline (PBS) (Sigma Aldrich, Poznań, Poland) (pH range 7.1–7.2). The lipase solution was changed every 48 h. Sterilization was conducted in a laminar chamber for 15 min, using UV light. Samples were placed in a 24-well plate. Each sample was covered with 1.5 mL of the solution. Three samples were used for each degradation period. Samples after degradation were dried in a vacuum dryer in 25 °C and then weighed. Mass loss after 4, 8, 12, 16, and 21 days was calculated using formula (3)

D = (m_0_ − m_1_)/m_0_ × 100% (3)

where D—mass loss [%], m_0_—sample mass before degradation [g], m_1_—sample mass after degradation [g].

## 3. Results and Discussion

**Figure 1:**
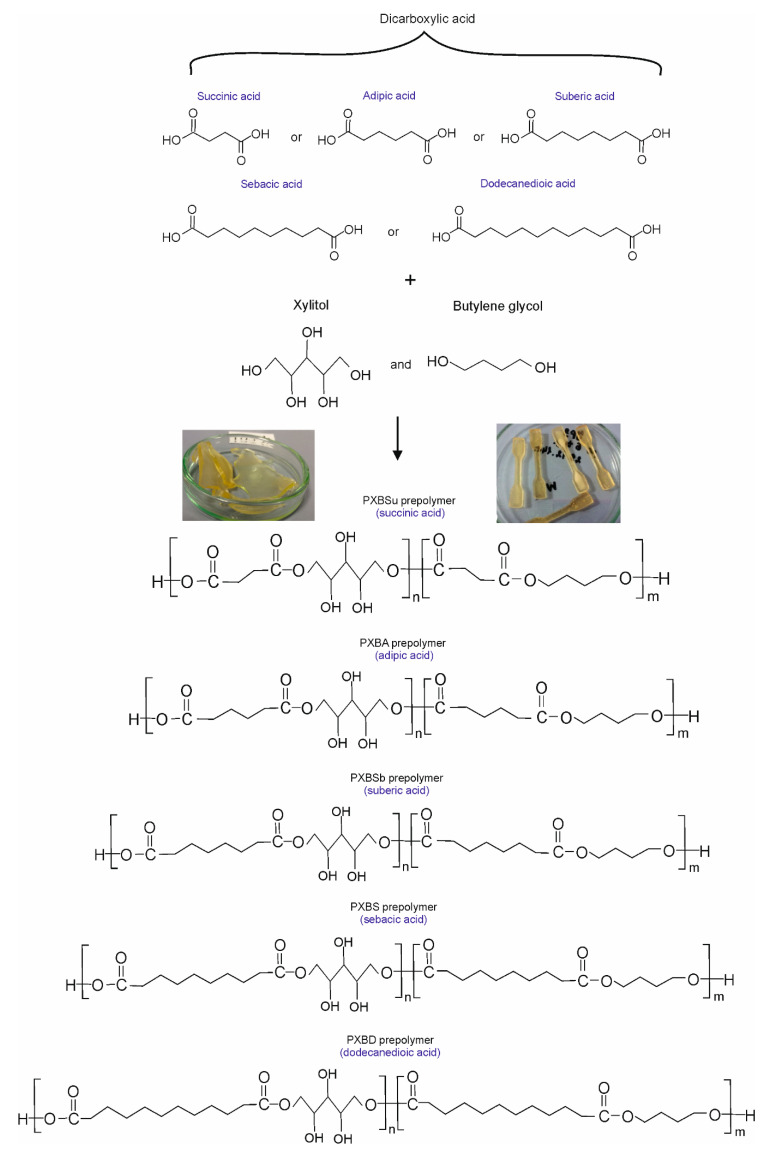
Scheme of poly(xylitol-dicarboxylate-co-butylene dicarboxylate) synthesis and structure.

The composition and properties of elastomers are summarized in Table 1, and a scheme of the structure is shown in Figure 1. 

### 3.1. Nuclear Magnetic Resonance Spectroscopy (NMR)

**Figure 2:**
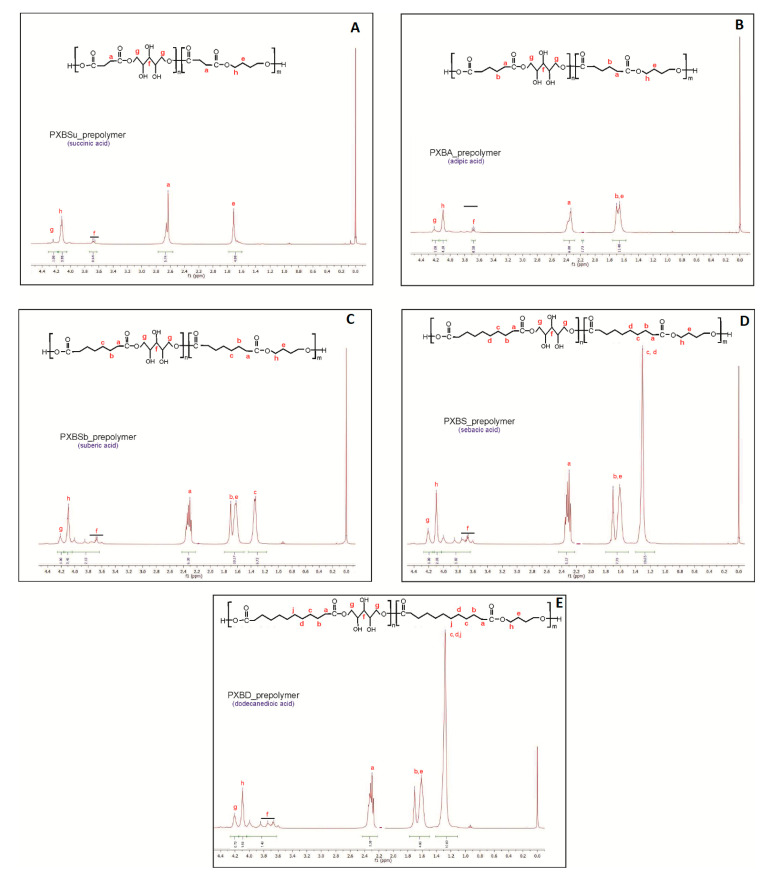
^1^H NMR of PXBSu(**A**), PXBA(**B**), PXBSb(**C**), PXBS(**D**), PXBD(**E**) prepolymers.

**Figure 3:**
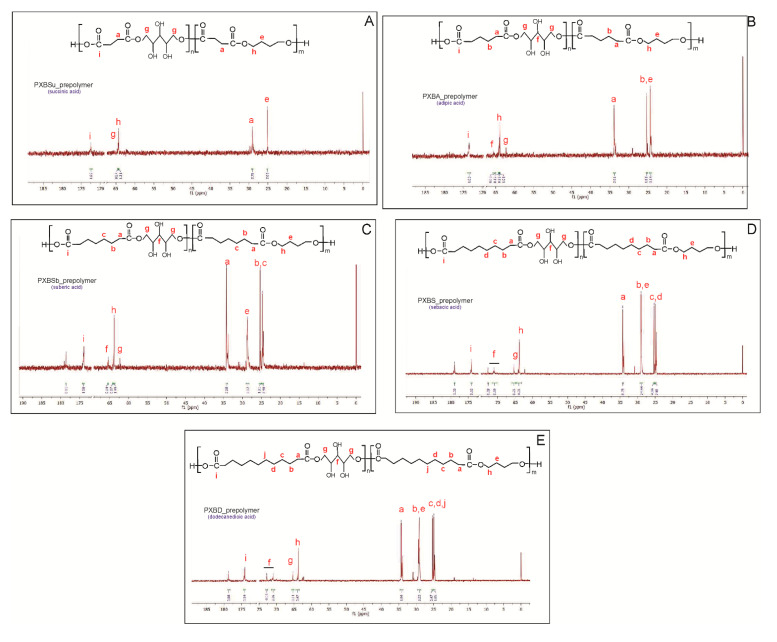
^13^C NMR of PXBSu(**A**), PXBA(**B**), PXBSb(**C**), PXBS(**D**), PXBD(**E**) prepolymers.

In order to analyze the chemical structure of the obtained polymers, and to confirm that the synthesis was successful, ^1^H NMR (Figure 2) and ^13^C NMR (Figure 3) analyses were performed. In ^1^H NMR peaks in the range of 1.2 to 2.8 ppm are due to CH_2_ groups in dicarboxylic acids and butylene glycol. Signals in the range of 3.6 to 4.0 ppm are due to CH_2_ groups in xylitol. 

The peak at about 4.1 ppm was attributed to the proton adjacent to the ester bond between dicarboxylic acid and butylene glycol, and the peak at about 4.2 ppm was ascribed to the proton adjacent to an ester bond between xylitol and dicarboxylic acid.

In ^13^C NMR, peaks in the range of 25 to 35 ppm are connected to carbon atoms in CH_2_ groups in dicarboxylic acids and butylene glycol. The peak at about 175 ppm was ascribed to carbon atoms in carbonyl groups. Two peaks at about 65 ppm are present—one is connected to carbon atoms next to an ester bond between dicarboxylic acid and butylene glycol, and the other is linked to a carbon atom adjacent to an ester bond between dicarboxylic acid and xylitol. 

### 3.2. Fourier Transform Infrared Spectroscopy (FTIR)

**Figure 4:**
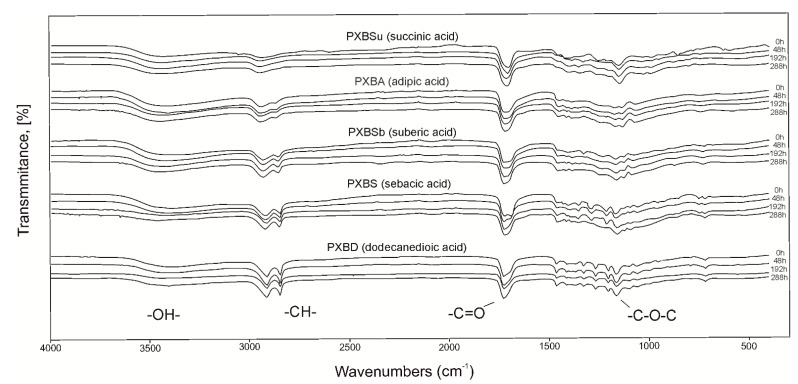
FTIR spectra of PXBSu, PXBA, PXBSb, PXBS, and PXBD prepolymers (0 h) and polymers at consecutive stages of cross-linking process (48 h, 144 h, 192 h and 288 h).

The FTIR spectra (Figure 4) show four peaks typical for sugar alcohol-based polyesters. The peak at 3450 cm^−1^ is connected to –OH groups (stretching), the peak at 2930 cm^−1^ is linked to –CH groups (asymmetrical, stretching vibration), the peak at 1730 cm^−1^ is associated with –C=O groups (asymmetrical, stretching), and the peak at 1170 cm^−1^ is related to –C–O–C groups (asymmetrical stretching). Due to –OH groups creating ester bonds during the cross-linking process, the intensity of the peaks at 3450 cm^−1^ is decreasing, and the intensity of the peaks at 1170 cm^−1^ is increasing with the progress of the cross-linking.

### 3.3. Thermal Properties: Differential Scanning Calorimetry (DSC) 

**Figure 5:**
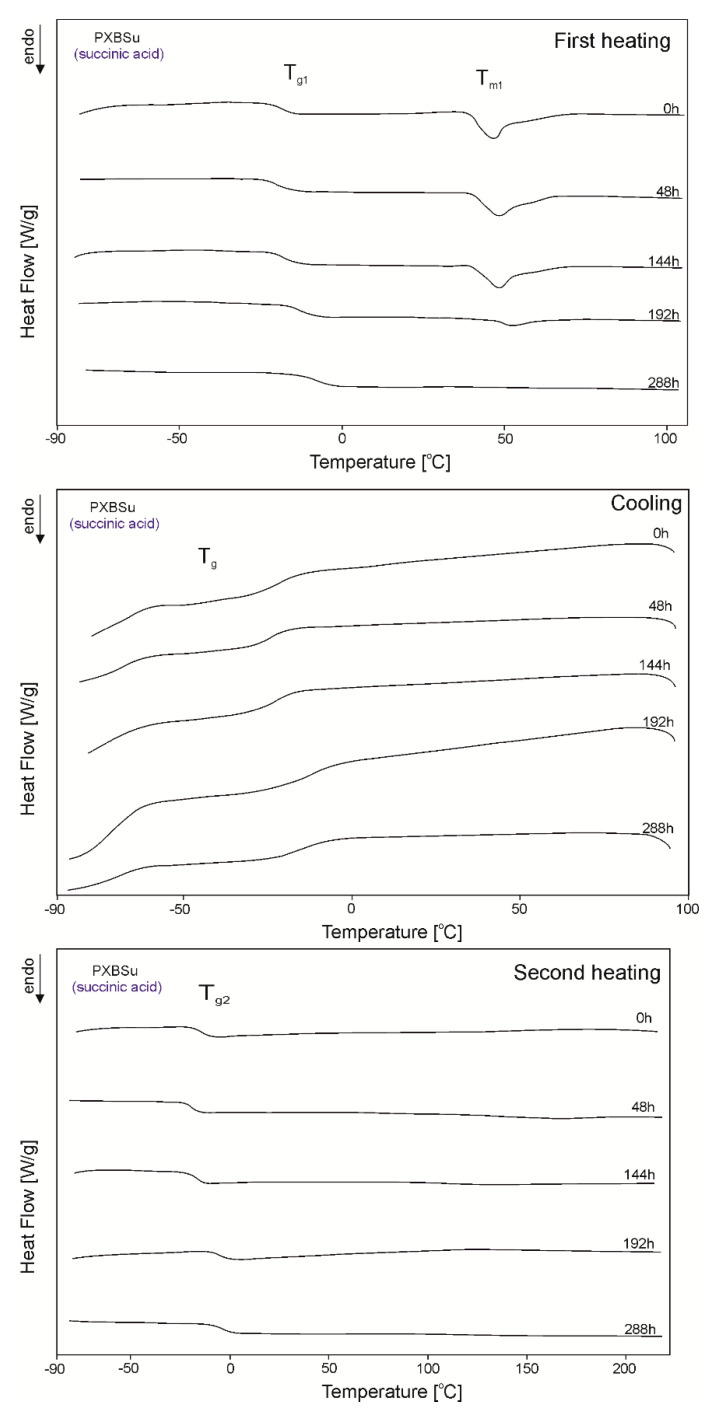
Differential scanning calorimetry (DSC) thermograms of PXBSu at the crosslinking stages subjected to first heating, second heating and cooling.

**Figure 6:**
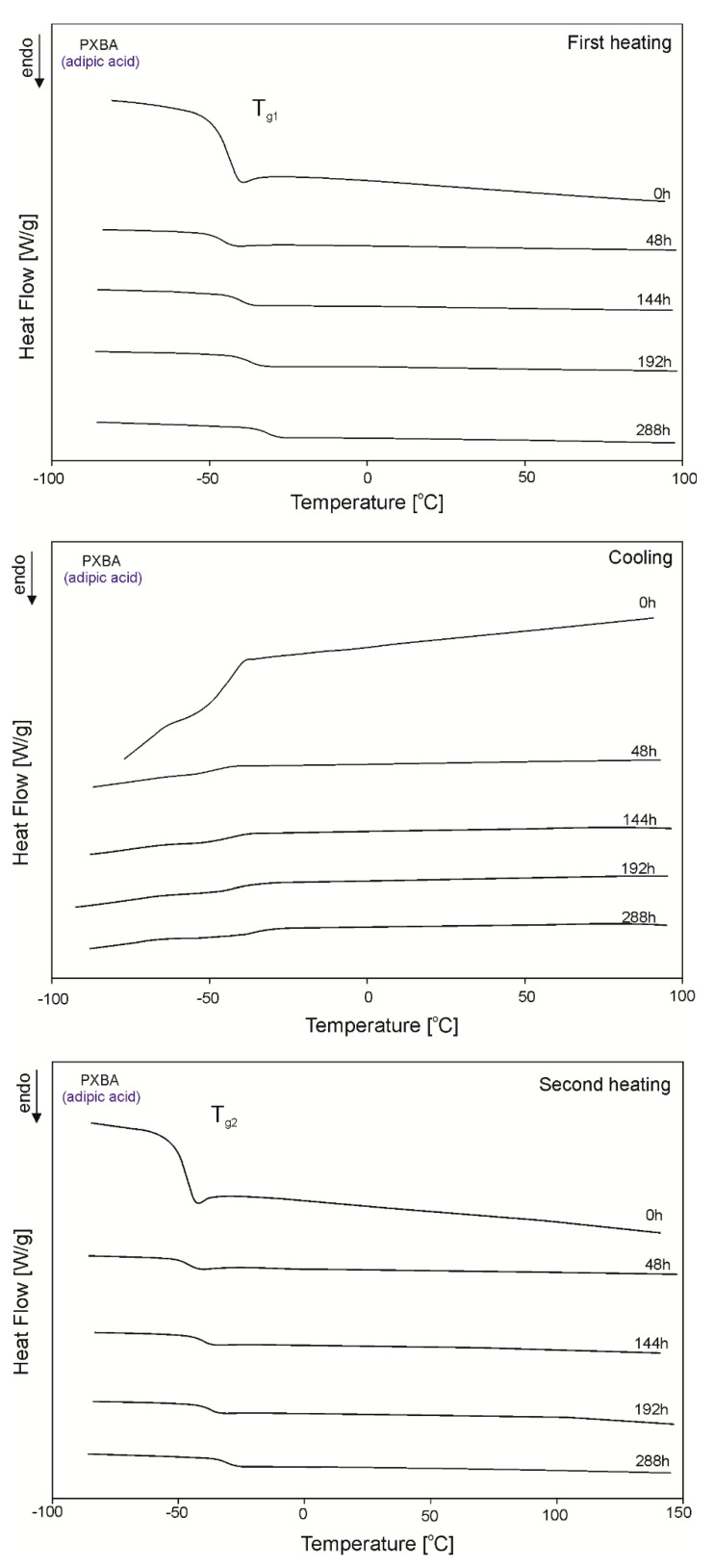
DSC thermograms of PXBA at the crosslinking stages subjected to first heating, second heating and cooling.

**Figure 7:**
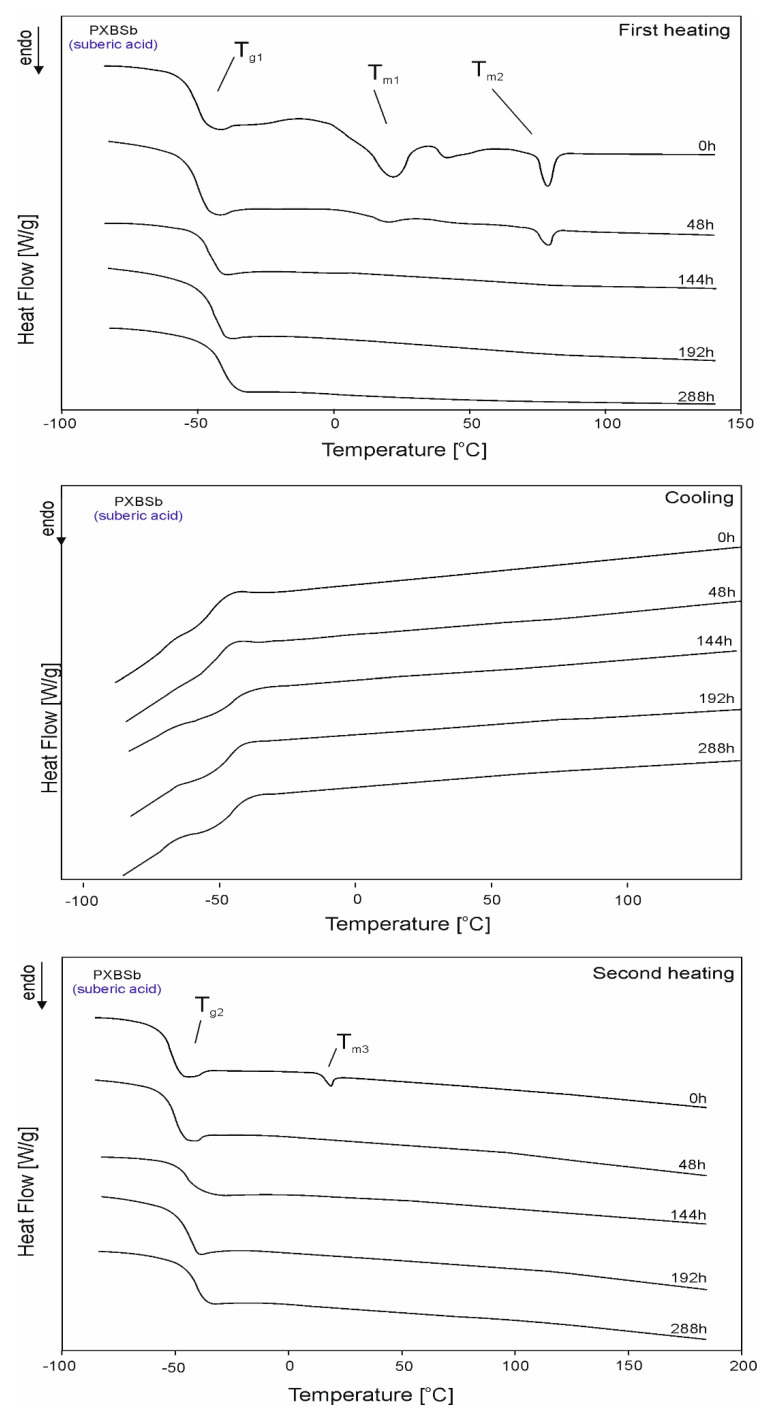
DSC thermograms of PXBSb at the crosslinking stages subjected to first heating, second heating and cooling.

**Figure 8:**
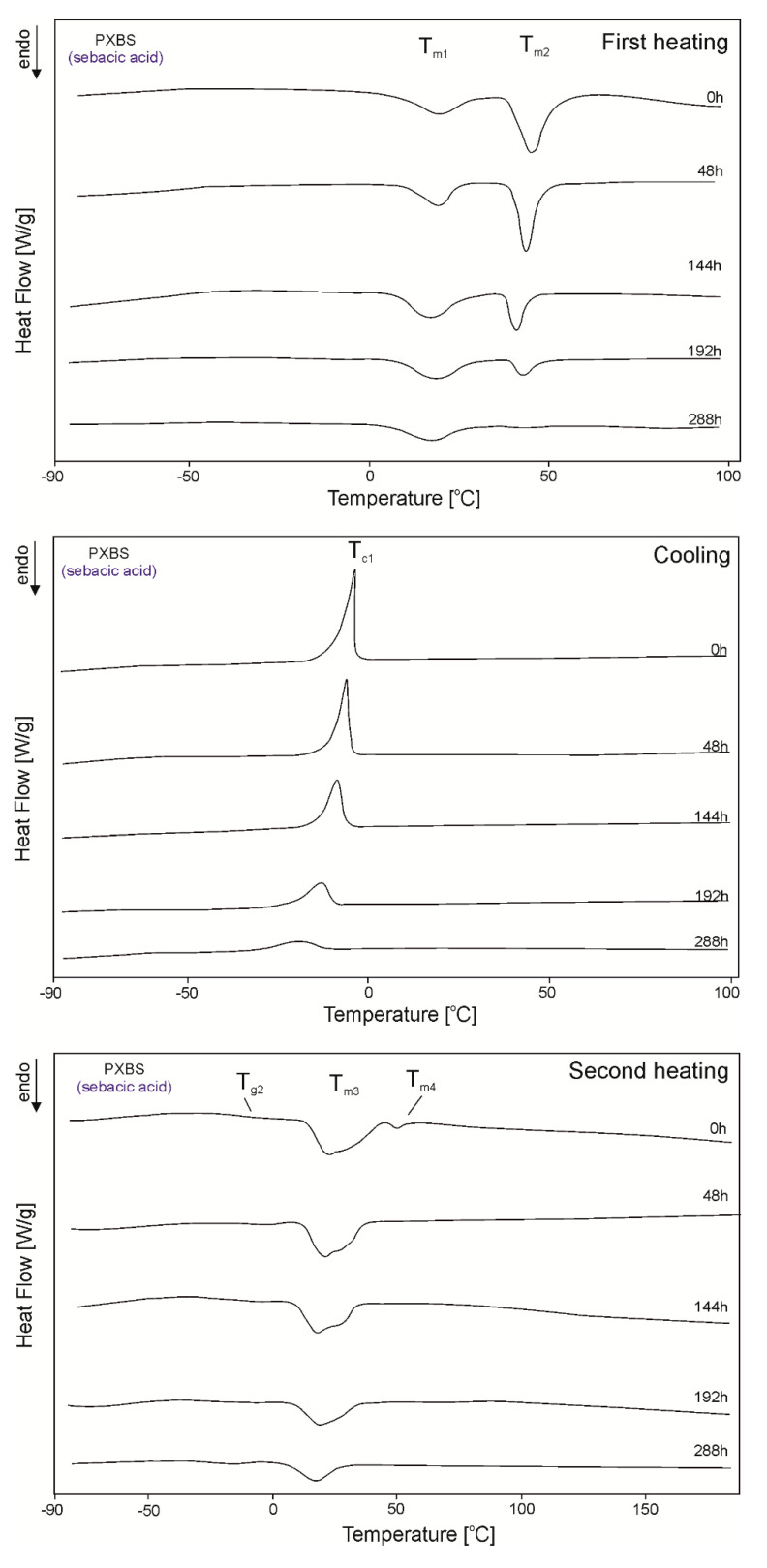
DSC thermograms of PXBS at the crosslinking stages subjected to first heating, second heating and cooling.

**Figure 9:**
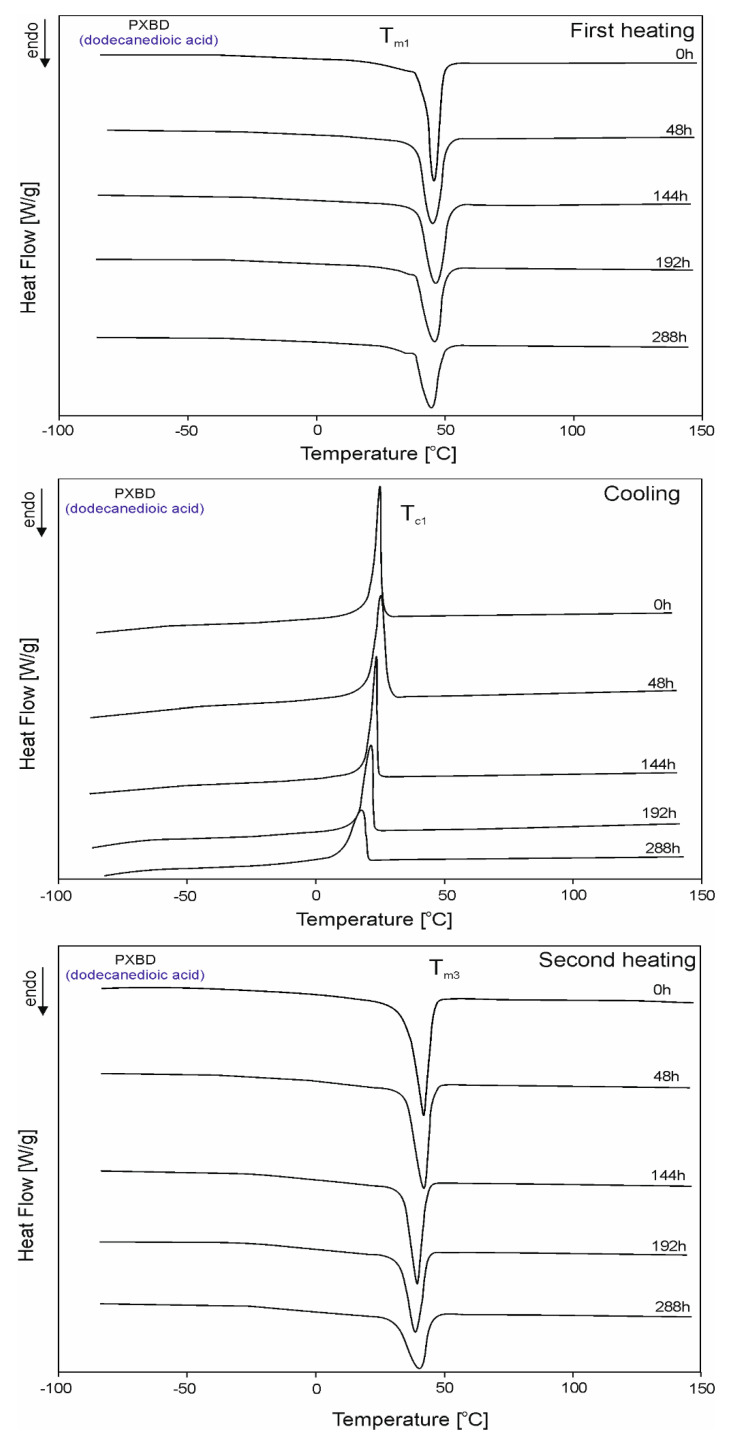
DSC thermograms of PXBD at the crosslinking stages subjected to first heating, second heating and cooling.

The DSC thermograms for PXBSu, PXBA, PXBSb, PXBS, and PXBD are shown in Figure 5, Figure 6, Figure 7, Figure 8 and Figure 9 and the thermal properties of the elastomers are given in Table 2.

A glass transition is observed for all polymers except PXBD. With cross-linking progress, the glass transition temperature (T_g_) increases, and the value of the change in heat capacity (ΔC_p_) during the glass transition decreases with the progress of the cross-linking process due to lower chain mobility, which confirms that cross-linking is indeed taking place. A melting transition is observed for all polymers except for PXBA, and its enthalpy decreases with the progress of the cross-linking process and the transition of the polymer structure into an amorphous system. Polymers with longer dicarboxylic acid chains have increased crystallinity, which is confirmed by the presence of two melting transitions with higher melting enthalpy (PXBSb and PXBS), and the much higher melting enthalpy exhibited by the PXBD polymer. Those results showing an increase in crystallinity also correlate with gel fraction test results. Two melting transitions displayed by PXBA and PXBSb are due to the melting of the poly(xylitol-dicarboxylate) block (T_m1_) and poly(butylene-dicarboxylate) block (T_m2_). A crystallization transition is only observable for two polymers with the longest dicarboxylic acid chains, and the highest crystallinity—PXBS and PXBD. The melting and crystallization transition being observable only for polymers based on dicarboxylic acids with the highest chain lengths is due to the crystalline structure becoming more ordered. PXBD is highly crystalline and it was not possible to determine the glass transition. 

### 3.4. Gel Fraction

**Figure 10:**
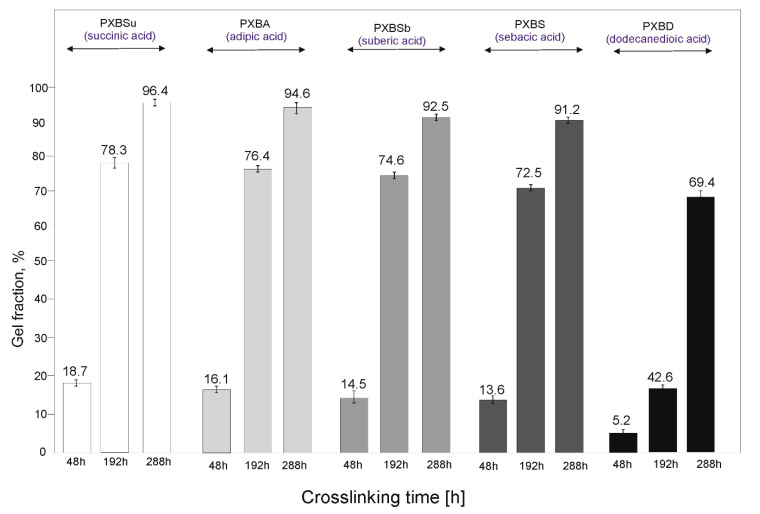
Gel fraction results for PXBSu, PXBA, PXBSb, PXBS and PXBD polymers.

Gel fraction (Figure 10) decreases with the length of the dicarboxylic acid chains due to the higher polymer crystallinity. The highest value is observed for the PXBSu polymer, and the lowest value is exhibited by the PXBD polymer. The gel fraction value increases at consecutive stages of the cross-linking process and the results correspond well to FTIR and DSC results. 

### 3.5. Mechanical Properties

**Figure 11:**
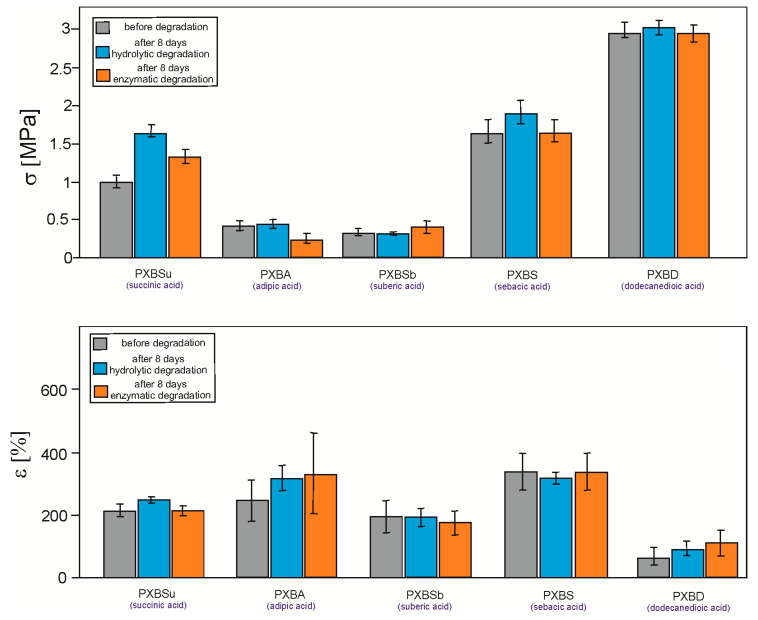
Mechanical properties of PXBSu, PXBA, PXBSb, PXBS and PXBD polymers.

Fully cross-linked materials (after 288h of cross-linking), before and after 8 days of degradation, were tested (Figure 11). The highest stress at break was exhibited by materials based on the longest dicarboxylic acids. The lowest stress at break was exhibited by PXBA and PXBSb materials. An interesting characteristic of all polymers is the increase in the value of elongation at break and stress at break for materials after 8 days of degradation, which is speculated to be due to the stabilization of the amorphous structure. PXBD is characterized by the highest stress at break and lowest elongation at break, due to its very high crystallinity, confirmed by both DSC and gel fraction analysis. Such a material no longer exhibits properties characteristic of elastomers.

### 3.6. Hydrolytic and Enzymatic Degradation

**Figure 12:**
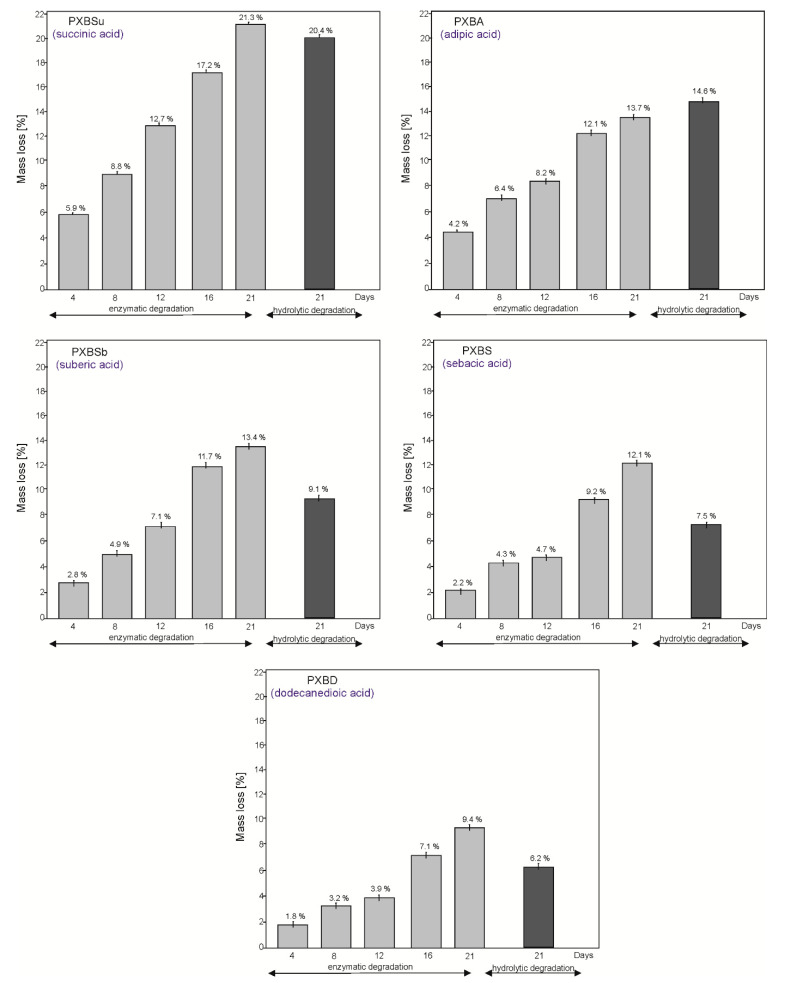
Hydrolytic and enzymatic degradation of PXBSu, PXBA, PXBSb, PXBS and PXBD polymers.

All the materials are biodegradable both enzymatically and hydrolytically (Figure 12). With the increase in the dicarboxylic chain length, the susceptibility to degradation decreases due to the higher crystallinity of the polymers. Higher crystallinity was confirmed by the DSC and gel fraction analysis. The polymers are more susceptible to enzymatic than hydrolytic degradation.

## 4. Conclusions

In this paper, poly(xylitol dicarboxylate-co-butylene dicarboxylate) materials based on dicarboxylic acids with different, even-numbered chain lengths are described. All the materials except PXBD have properties typical for elastomers. Successful synthesis was confirmed by both ^1^H NMR, ^13^C NMR and FTIR analyses. By performing gel fraction tests and DSC analysis, the best cross-linking time was determined to be 288h. The progress of cross-linking at different stages was confirmed by the increase in the gel fraction value, the decrease in melting enthalpies and the decrease in peak intensities related to –OH groups, as well as the increase in peak intensities linked to –C–O–C groups as determined by FTIR. Degradation times and mechanical properties can be tailored for specific applications by using different dicarboxylic acids. With respect to tailoring the properties, it is better to change the acid chain length than the hydroxyl group content [23], since a change in properties obtained by changing the acid chain length is much more predictable. Possible future research could include the synthesis of an even wider variety of alditol-based polyesters with either different glycols or odd-numbered dicarboxylic acids.

## Figures and Tables

**Table 1:** Composition and selected properties of poly(xylitol succinate-*co*-butylene succinate) (PXBSu), poly(xylitol adipicate-*co*-butylene adipicate) (PXBA), poly(xylitol suberate-*co*-butylene suberate) (PXBSb), poly(xylitol sebacate-*co*-butylene sebacate) (PXBS), poly(xylitol dodecanedioate -*co*-butylene dodecanedioate) (PXBD).

Polymer Sample	Molar Composition [mol]			Molar Composition Determined by ^1^H NMR [mol]		E_ 50% [MPa]	E_ 100% [MPa]	σ_r_ [MPa]	ε_r_ [%]	H *ShA*
	SuA	XL	BG	XL	BG					
PXBSu (succinic acid)	2	1	1	1.0	3.95	0.469 +/−0.04	0.403 +/−0.03	0.991 +/−0.13	214 +/15.51	46.4 +/−0.42
	AA	XL	BG	XL	BG					
PXBA (adipic acid)	2	1	1	1.0	4.10	0.165 +/− 0.04	0.148 +/−0.04	0.408 +/−0.09	248 +/67.55	36.8 +/−0.38
	SbA	XL	BG	XL	BG					
PXBSb (suberic acid)	2	1	1	1.0	3.41	0.212 ± 0.082	0.188 ± 0.07	0.328 ± 0.05	196 +/51.73	44.9 +/−0.34
	SA	XL	BG	XL	BG					
PXBS (sebacic acid)	2	1	1	1.0	2.33	0.756 ± 0.04	0.605 ± 0.03	1.632 ± 0.28	339 +/59.18	35.3 +/−0.45
	DA	XL	BG	XL	BG					
PXBD (dodecanedioic acid)	2	1	1	1.0	2:14	1.945 ± 0.08	1.876 ± 0.05	2.995 ± 0.35	42 +/24.16	28.6 +/−0.31

Stress at break (σ_r_), elongation at break (ε_r_), modulus at 50% elongation (E_50%), modulus at 100% elongation (E_100%), hardness (H), succinic acid (SuA), adipic acid (AA), suberic acid (SbA), sebacic acid (SA), dodecanedioic acid (DA), butylene glycol (BG), xylitol (XL).

**Table 2:** Thermal properties of the PXBSu, PXBA, PXBSb, PXBS, PXBD.

	First Heating						Cooling		Second Heating					
Cross−Linking Time	T_g1_ [°C]	∆c_p_ [J/g°C]	T_m1_ [°C]	∆H_m1_ [J/g]	T_m2_ [°C]	∆H_m2_ [J/g]	T_c1_ [°C]	∆H_c1_ [J/g]	T_g2_ [°C]	∆c_p_ [J/g°C]	T_m3_ [°C]	∆H_m3_ [J/g]	T_m4_ [°C]	∆H_m4_ [J/g]
	*PXBSu (succinic acid)*													
0 h	−19.9	0.522	46.8	15.41	-	-	−26.4	0.84	−15.7	0.57	-	-	-	-
48 h	−18.6	0.533	48.4	13.92	-.	-	−24.8	0.542	−18.8	0.651	-	-	-	-
144 h	−17.6	0.596	48.5	12.32	-	-.	−22.5	0.668	−17.5	0.719	-	-	-	-
192 h	−12.2	0.449	50.9	7.77	-.	-	−18.9	0.897	−5.2	0.475	-	-	-	-
288 h	−7	0.572	n.o.	n.o.	-	-	−18.6	0.624	−2.8	0.543	-	-	-	-
	*PXBA (adipic acid)*													
0 h	−46.3	0.983	-	-	-	-	-	-	−44.6	0.975	-	-	-	-
48 h	−45.6	0.705	-	-	-	-	-	-	−44.9	0.709	-	-	-	-
144 h	−40.3	0.677	-	-	-	-	-	-	−40.4	0.690	-	-	-	-
192 h	−37.5	0.652	-	-	-	-	-	-	−37.6	0.648	-	-	-	-
288 h	−30.9	0.592	-	-	-	-	-	-	−30.6	0.557	-	-	-	-
	*PXBSb (suberic acid)*													
0 h	−50.8	0.676	20.4	7.32	78.8	2.41	-	-	−52.7	0.738	18.7	0.65	-	-
48 h	−50.1	0.740	19.3	0.95	78.9	1.13	-	-	−50.4	0.757	-	-	-	-
144 h	−45.9	0.553	-	-	-	-	-	-	−44.2	0.526	-	-	-	-
192 h	−44.6	0.670	-	-	-	-	-	-	−46.1	0.642	-	-	-	-
288 h	−41.6	0.624	-	-	-	-	-	-	−41.4	0.656	-	-	-	-
	*PXBS (sebacic acid)*													
0 h	-	-	19	24.17	44.9	50.89	−3.9	65.87	−13.1	0.558	22.6	61.3	50.7	3.17
48 h	-	-	19.5	23.28	43.5	41.56	−6.3	47.16	−12.8	0.128	21.1	53.15	-	-
144 h	-	-	17.2	34.1	41	18.18	−8.8	42.77	−19.3	0.426	18.3	48.41	-	-
192 h	-	-	18.9	28.67	42.9	9.76	−13.3	30.3	−20.5	0.264	19.9	35.52	-	-
288 h	-	-	17.5	24.01	41.2	0.66	−19	15.87	−28	0.248	17.6	23.18	-	-
	*PXBD (dodecanedioic acid)*													
0 h	-	-	45.6	83.43	-	-	24.8	65.43	-	-	41.8	74.92	-	-
48 h	-	-	44.2	80.80	-	-	25.3	72.71	-	-	41.9	65.60	-	-
144 h	-	-	46.3	75.23	-	-	23.5	55.16	-	-	38.2	54.83	-	-
192 h	-	-	45.6	67.79	-	-	21.5	52.23	-	-	38.7	51.73	-	-
288 h	-	-	44.7	57.92	-	-	17.6	44.35	-	-	40.4	40.92	-	-

T_g1_ and T_g2_—glass transition temperatures; ∆c_p_—change in the heat capacity during glass transition at T_g1_ and T_g2_; T_m1_, T_m2_, T_m3_ and T_m4_—melting temperature; T_c1_—crystallization temperatures; ΔH_m1_, ΔH_m2_, ΔH_m3_ and ΔH_m4_—heat of melting at T_m1,_ T_m2_, T_m3_ and T_m4_; ΔH_c1_—crystallization heat in T_c1_.
